# Supplementary material for: “The impact of the Little Orange Book on how parents/carers manage symptoms of illness in children: A mixed methods study”
Source: PLoS One. 2025 Jan 22;20(1):e0295470. doi: 10.1371/journal.pone.0295470 (PMC11753658; doi:10.1371/journal.pone.0295470)
Supplement: S1 File — (DOCX) [file pone.0295470.s001.docx]

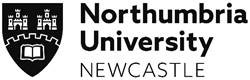

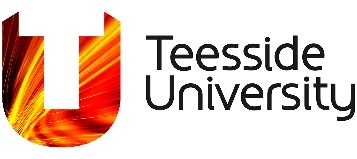

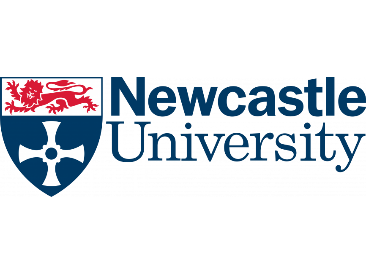


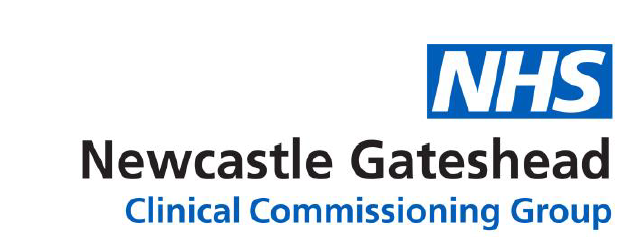


# PAGE 1: PARTICIPANT INFORMATION SHEET

Project title: Evaluation of healthcare information given to new parents (Little Orange Book)

INVITATION

You are invited to take part in a survey about your experience of using the revised edition of The Little Orange Book. The Little Orange Book is an information book given to parents and carers of children in Newcastle and Gateshead. It contains information and advice about using health care services for your child/children. We would like to hear your views if you have received The Little Orange Book from and including 2018. The study has been given ethical approval to go ahead by Northumbria University.

ABOUT US

We are a group of researchers based across the North East at Northumbria, Newcastle and Sunderland Universities and this study has been funded by the Newcastle Gateshead Clinical Commissioning Group (NGCCG).

WHY IS THIS STUDY BEING CARRIED OUT?

The study is being carried out to help understand your experiences of using the revised edition of The Little Orange Book. Your views will also be used to help improve The Little Orange Book in the future.

WHY HAVE I BEEN INVITED?

You have been invited to take part because we understand that you are a parent/carer living in Newcastle or Gateshead who has received a copy of the revised edition of The Little Orange Book from 2018 onwards.

DO I HAVE TO TAKE PART IN THIS STUDY?

No - you do not have to take part in this study. Your decision will not be shared with anyone outside of the research team and it will not influence any of the health care services you receive. Please read all sections of this information sheet before making a decision.

If you have any concerns or would like to talk to someone to help you decide whether or not you would like to participate in the study, you may contact:

Dr.Lynette Shotton (project lead) – lynette.shotton@northumbria.ac.uk

Dr Amy Johnson (Senior Research Assistant) – [amy6.johnson@northumbria.ac.uk](mailto:amy6.johnson@northumbria.ac.uk)

If you have any concerns about the study and would like to talk to someone at Northumbria University who knows about, but is not involved in, the study please contact Dr Se Kwang Hwang ([sekwang.hwang@northumbria.ac.uk](mailto:sekwang.hwang@northumbria.ac.uk)), Northumbria University Ethics Lead for the department of Social Work, Education and Community Wellbeing.

WHAT WILL TAKING PART INVOLVE?

If you decide to complete this survey, you will be asked questions about your experience of using the revised edition of The Little Orange Book, your use of children’s healthcare services and how you feel the revised edition of The Little Orange Book can be improved. You will be asked to provide some personal information such as your ethnicity, gender and sexuality. This will be used to help us describe the group of people who have completed the survey. All the details you give will be confidential. We expect the survey to take around 20 minutes to complete. When you finish the survey, you will be directed to a debrief page which will give you information about advice and support if you have been affected by any issues in this questionnaire. At the end of the survey, you will have the opportunity to enter into a draw to win a Highstreet voucher worth £25.

You will also be asked if you would be willing to be contacted for a follow-up interview to further talk about your experiences. Interviews can be focus groups or individual interviews and will be audio recorded. If you are interested in taking part in the interview, we will ask you for contact details and (if you would like to take part in an interview) a preferred time and method of contact. People taking part in the interview study will be given an additional information sheet describing the interview process.

WHO CAN TAKE PART IN THIS STUDY?

To take part in this survey:

- You must be 18 years or over.
- You must be a parent, carer or guardian (including grandparents).
- You must have been given a copy of The Little Orange Book after 2018.
- You must live (or used to live) in the North East of England in Newcastle or Gateshead at the time you were given a Little Orange Book.
- You must be able to give your consent to participate in the evaluation and fully understand what you are being asked to do and what will happen.

WILL MY TAKING PART BE KEPT CONFIDENTIAL?

Yes, the data that you give in this survey is anonymous. If you choose to take part in an individual interview or focus group about your views and experiences using the Revised Edition of The Little Orange Book, you will be asked to provide contact details. This is so that the research team can get in touch with you to organise the interview and these contact details will not be stored with the data from the survey. Only the research team will have access to this information and this will be destroyed once the project is completed.

Before deciding to take part in the interview, you must be aware that complete anonymity cannot be guaranteed. If you disclose any information that raises concerns about your welfare or that of anyone else, the research team may have to escalate this to meet safeguarding legislation. In the first instance, Dr Lynette Shotton would escalate the concern if appropriate and raise with relevant organisations.

When completing this survey, you also have the opportunity to enter into a prize draw to win a Highstreet voucher and will be asked to provide contact details. This is so we can get in touch with you if you win. Again, your name or contact details will not be stored with the information you give in this survey.

WHERE AND FOR HOW LONG WILL MY DATA BE STORED?

The research team will only gather personal data from you for the purpose of this study. The information you provide will be stored and used carefully to meet the General Data Protection Regulation and the Data Protection Act (2018). Your survey will be stored on a password protected, secure university computer system. Any paper survey responses will be scanned and stored on this password protected computer system. The paper copies will then be destroyed. The surveys will be anonymised to protect your identity. Your survey responses will be kept for one year following completion of the evaluation in case further analysis is needed. Evaluation data will be retained until June 2023.

WHAT ARE THE RISKS OF PARTICIPATING IN THIS STUDY?

We do not consider any specific risks of taking part in the survey. The survey will take around 20 minutes of your time. Some of the questions will ask you about your experiences of using health services to help you when your child/children has been ill. These may be difficult or upsetting for you to answer. You do not have to answer any questions that you do not want to and you can also select the ‘prefer not to say’ option.

WHAT SUPPORT WILL BE AVAILBLE TO ME?

If you feel that you need support and have been affected by any issues raised during participation in the study, the following registered charity are able to offer services to support you:

Samaritans Telephone: 116 123 (UK) / Website: www.samaritans.org.

You are able to call Samaritans for free, at any time, from any phone.

If you have immediate concerns about your wellbeing, please seek medical attention from your general practitioner or alternative health care provider. If you require out of hours medical care, you are able to contact NHS 111 for assistance or dial 999 in an emergency situation.

CAN I WITHDRAW FROM THE STUDY AND DO I HAVE TO TAKE PART?

You will be asked to provide a unique identifier for this study. You are able to withdraw from the study at any time up to 7 days following completion of this survey by providing the research team with your unique identifier. If you would like to withdraw, you can contact Dr Lynette Shotton ([Lynette.shotton@northumbria.ac.uk](mailto:Lynette.shotton@northumbria.ac.uk)) or Dr Amy Johnson ([amy6.johnson@northumbria.ac.uk](mailto:amy6.johnson@northumbria.ac.uk)). Withdrawal from the study will not impact on any of your legal rights or your/your child/children’s medical care. After the 7 days has passed, you may no longer withdraw your data from the study.

WHAT HAPPENS NEXT?

If you are happy to continue with the study, please read and complete the online consent form and you will be directed to the survey.

*Personal data including special category data obtained for the purposes of this research project is processed lawfully in the necessary performance of scientific or historical research or for statistical purposes carried out in the public interest. Processing of personal data including special category data is proportionate to the aims pursued, respects the essence of data protection and provides suitable and specific measures to safeguard the rights and interests of the data subject in full compliance with the General Data Protection Regulation and the Data Protection Act 2018.*

# PAGE 2: CONSENT FORM

Lynette Shotton (Northumbria University), Matthew Breckons (Newcastle University), Kathryn Carruthers (Teesside University), Sunil Bhopal (Newcastle University), Floor Christie De Jong (University of Sunderland) and Judith Rankin (Newcastle University) and Amy Johnson (Northumbria University)

Thank you for your interest in participating in the Little Orange Book evaluation online survey to explore the experiences of parents and carers who have received and/or used the revised edition of the Little Orange Book. To participate in the evaluation, you must provide your consent. If you would like to take part in this study, please read the statements below and click ‘I agree’.

- I confirm that I have read the participant information sheet dated for the Little Orange Book online Survey. I have had the opportunity to consider the information, ask questions and have had these answered satisfactorily.
- I understand that my participation is voluntary and that I am free to withdraw at any time up to 7 days following the survey without giving any reason and without any medical care or legal rights being affected. After 7 days I will be unable to withdraw this data.
- I agree to provide information to the investigator and understand that my contribution will remain confidential.
- I understand that after I participate I will receive a debrief form providing me with information about the study and contact details for the researcher.
- I understand that the information collected about me may be used to support the design of other research and teaching in the future and may be shared anonymously with other researchers.
- I agree to take part in the above online survey

I agree:

1. Please give a unique identifier here. This is a unique code that you choose. Please do not include any personal information, such as names. If you would like to withdraw from the study (within 7 days of taking part), please contact the research team and provide this identifier. (open response)

# PAGE 3: SCREENING QUESTIONS

The Little Orange Book provides information to help decide if and what type of services to access when children are unwell. This was first released in 2016 and was updated in 2018 (which we refer to as ‘The Revised Edition of The Little Orange Book’). We are interested in hearing your views about **the Revised Edition of The Little Orange Book**, such as how you used this and if you think it could be improved.

The Revised Edition of The Little Orange Book will look like this:


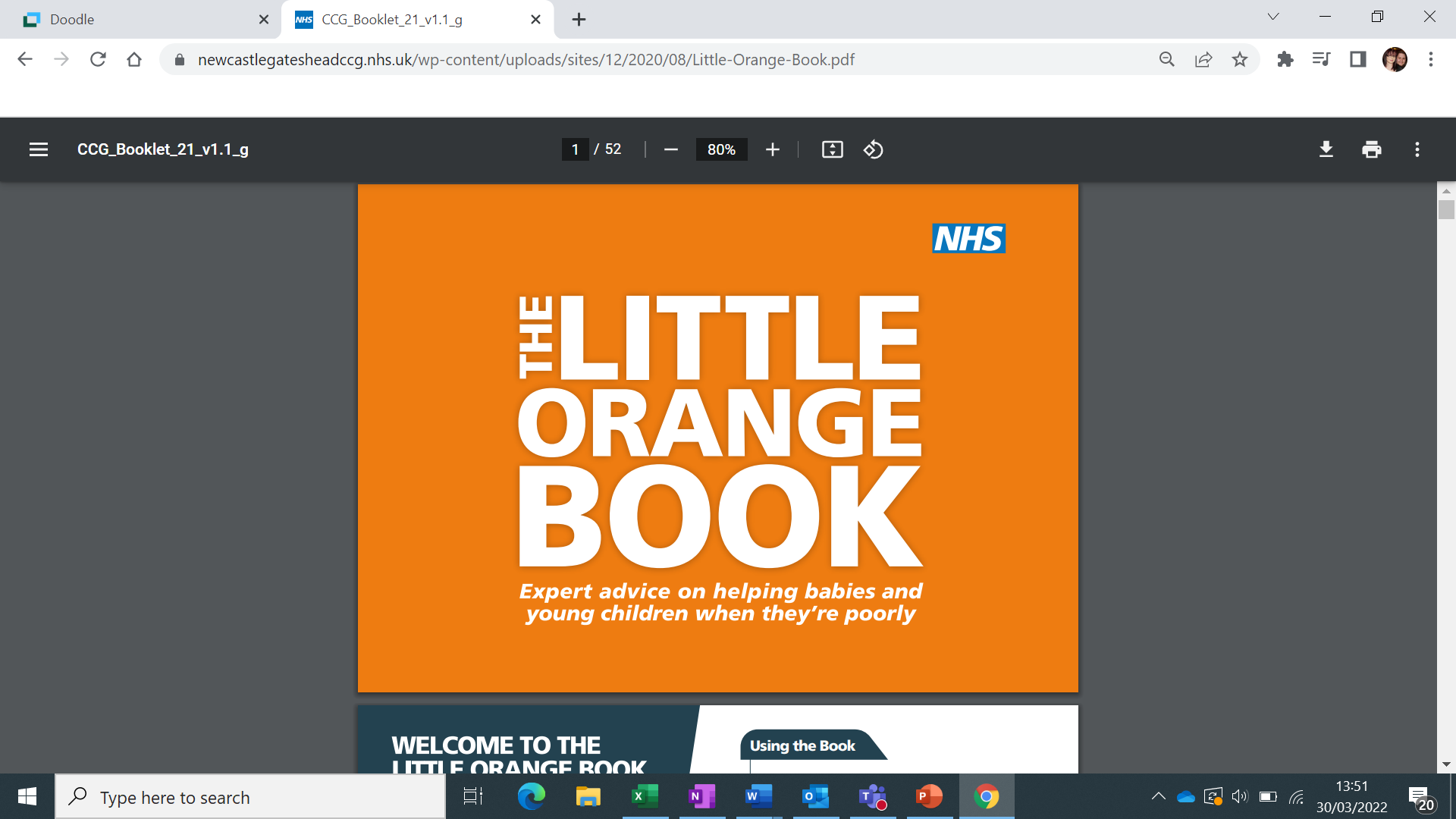


It also includes the date on the bottom right corner of the last page:


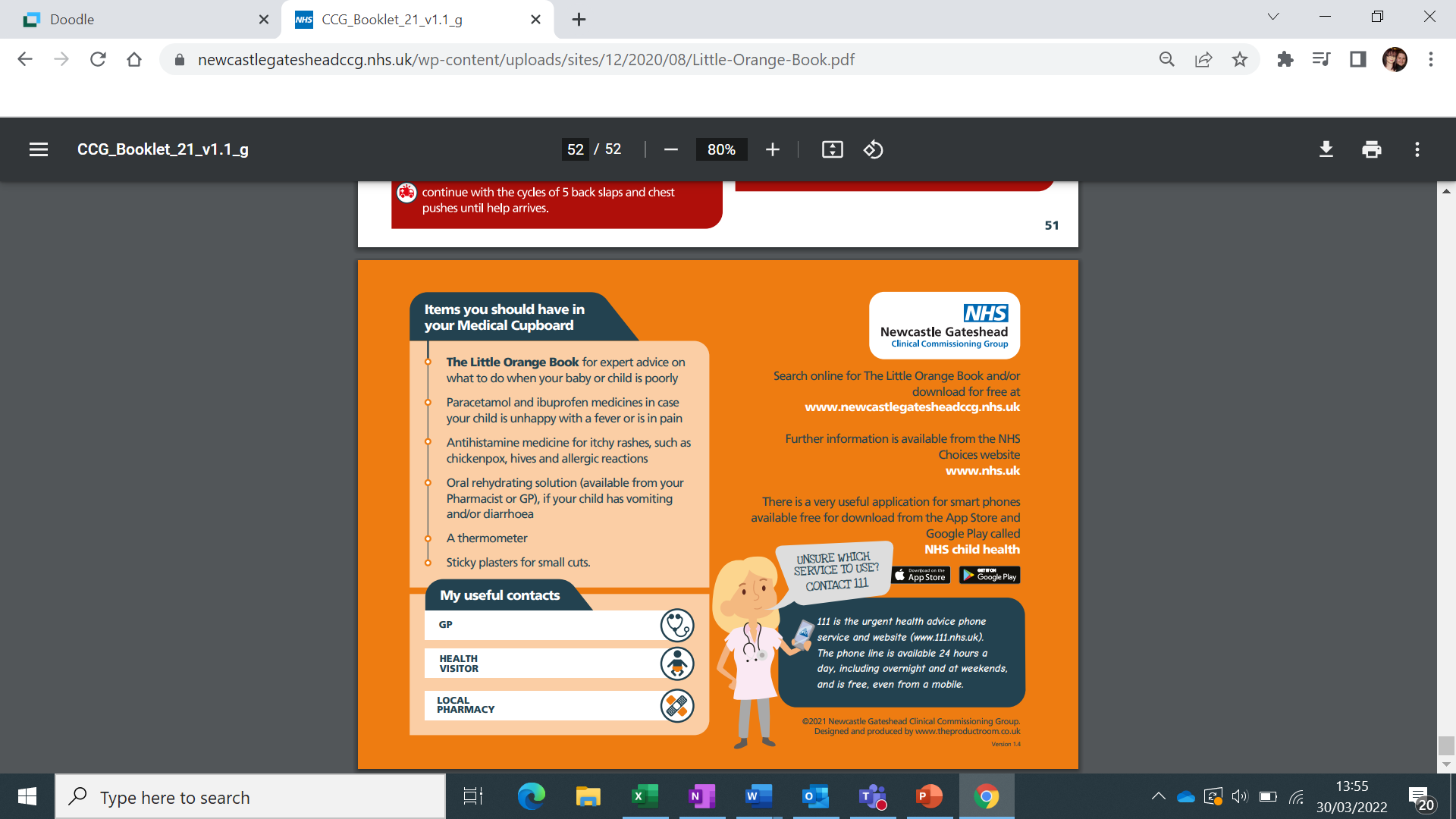


If this date is after 2018, you have the revised edition of The Little Orange Book.

1. Have you ever been given a copy of the revised edition of the Little Orange Book? The revised edition of The Little Orange Book was given out **from 2018 onwards**. The revised edition of The Little Orange Book is shown in image 2 (drop down menu)

- Yes
- No

i. When did you receive your copy of The Little Orange Book?

- 2018-2020
- 2021-2022

1. Have you accessed an online edition of The Little Orange Book? (drop down menu)
   - Yes
   - No

LOGIC – IF ANSWERING NO TO Q2, Q3 AND Q4, DIRECT PAGE 6. IF ANSWERING YES, GO TO PAGE 4.

# PAGE 4: THE REVISED EDITION OF THE LITTLE ORANGE BOOK

The Little Orange Book provides information to help decide if and what type of services to access when children are unwell. We are interested in hearing your views about **the Revised Edition of The Little Orange Book**, such as how you used this and if you think this could be improved.

1. Where did you first get a copy of the revised edition of The Little Orange Book? Please select ONE answer.

- Health appointment in the community (e.g. GP surgery/Walk in centre/Midwife/Health Visitor etc.)
- Health appointment in a hospital (e.g. A+E, outpatients etc.)
- My child’s school/nursery/childcare setting
- Community centre/Children centre/organisation
- A charity
- Accessed this online
- Family member/friend
- Other (please state)
- Can’t remember
- Prefer not to say

1. Have you used your copy of the revised edition of The Little Orange Book to help make healthcare decisions for your child/children when they were unwell? (drop down menu)

- Yes
- No
- Prefer not to say

LOGIC – IF YES, GO TO Q7. IF NO, GO TO Q6i

i. What stopped you from using the revised edition of The Little Orange Book? (open response)

ii. What would encourage you to use the revised edition of The Little Orange Book? (Open response)

iii. If you haven’t used the revised edition of The Little Orange Book, where did you did you go for advice?

- Health professionals in the community (e.g. GP/Midwife/Health Visitor etc.)
- Health professionals in a hospital (e.g. A+E, outpatients etc.)
- My child’s school/nursery/childcare setting
- Community centre/Children centre/organisation
- A charity
- Found advice online
- Family member/friend
- Previous edition of The Little Orange Book (before 2018)
- Other (please state)

7. What is your relationship to the child/children for whom you received or used the revised edition of the Little Orange Book? (drop down menu)

- - Parent/Guardian
  - Grandparent
  - Stepparent
  - Foster parent
  - Other (please state)
  - Prefer not to say

8. What is the year of birth of your child/children for whom you have consulted the revised edition of the Little Orange Book? (Please select all that apply)

|  | Before 2010 | 2010-2014 | 2015-2017 | 2018-2020 | 2021-2022 | I have not used the revised edition of The Little Orange Book | Prefer not to say |
| --- | --- | --- | --- | --- | --- | --- | --- |
| First Child |  |  |  |  |  |  |  |
| Second Child |  |  |  |  |  |  |  |
| Third Child |  |  |  |  |  |  |  |
| Fourth Child |  |  |  |  |  |  |  |

9. What was your postcode when you received the revised edition of the Little Orange Book? Please only give the first FOUR DIGITS (e.g. NE12) (Open text response)

10. Do you live at the same postcode?

- Yes
- No
- Prefer not to say

LOGIC – IF NO, GO TO Q10i

i. If no, what is your current postcode?

Please rate to what extent you agree with the following statements.

11. The revised edition of the Little Orange Book has made me *more likely to access non-emergency healthcare services* (e.g. GP, health visitor, midwife etc.).

- Strongly agree
- Agree
- Neither agree nor disagree
- Disagree
- Strongly disagree

12. The revised edition of the Little Orange Book has made me *less likely* *to access emergency healthcare services* (e.g. A&E or Urgent Care Centre)

- Strongly agree
- Agree
- Neither agree nor disagree
- Disagree
- Strongly disagree

13. The revised edition of The Little Orange Book has increased my confidence to use health services appropriately

- Strongly agree
- Agree
- Neither agree nor disagree
- Disagree
- Strongly disagree

14. The revised edition of The Little Orange Book has increased my confidence to request same day appointments with my GP

- Strongly agree
- Agree
- Neither agree nor disagree
- Disagree
- Strongly disagree

15. Using the revised edition of the Little Orange Book has helped me to decide whether my child/children should attend their nursery/school/childcare setting

- Strongly agree
- Agree
- Neither agree nor disagree
- Disagree
- Strongly disagree

16. Using the revised edition of the Little Orange Book has helped me to decide whether my child/children should attend activities or social gatherings outside of their educational or childcare setting

- Strongly agree
- Agree
- Neither agree nor disagree
- Disagree
- Strongly disagree

17. The revised edition of The Little Orange Book has increased my confidence in caring for my child/children when they are unwell

- Strongly agree
- Agree
- Neither agree nor disagree
- Disagree
- Strongly disagree

18. Has the revised edition of the Little Orange Book affected your decision to access healthcare services?

- Yes
- No
- Prefer not to say

LOGIC – IF YES, GO TO 18i

i. If yes, please explain: (open response)

19. In what ways, if any, has the revised edition of the Little Orange Book affected the way in which you have sought medical or health care advice for the child/children you care for? (Open text box)

# PAGE 5: YOUR VIEWS OF THE REVISED EDITION OF THE LITTLE ORANGE BOOK

We would now like you to share your opinions of the revised edition of The Little Orange Book. Please tell us the extent to which you agree with the following statements.

20. My healthcare professional explained how to use the revised edition of The Little Orange Book

- Strongly agree
- Agree
- Neither agree nor disagree
- Disagree
- Strongly disagree
- Prefer not to say

21. The revised edition of The Little Orange Book was easy to understand

- Strongly agree
- Agree
- Neither agree nor disagree
- Disagree
- Strongly disagree
- Prefer not to say

22. The layout of the revised edition of The Little Orange Book was useful

- Strongly agree
- Agree
- Neither agree nor disagree
- Disagree
- Strongly disagree
- Prefer not to say

24. The range of topics included in the revised edition of The Little Orange Book were relevant

- Strongly agree
- Agree
- Neither agree nor disagree
- Disagree
- Strongly disagree
- Prefer not to say

25. The Red Amber Green colour guide helped me make decisions

- Strongly agree
- Agree
- Neither agree nor disagree
- Disagree
- Strongly disagree

26. The photographs and images in the revised edition of The Little Orange Book were useful

- Strongly agree
- Agree
- Neither agree nor disagree
- Disagree
- Strongly disagree

27. I would like more pictures and images in the revised edition of The Little Orange Book.

- Strongly agree
- Agree
- Neither agree nor disagree
- Disagree
- Strongly disagree

28. I would recommend the revised edition of The Little Orange Book to other parents or carers of young children

- Yes
- No
- Prefer not to say

LOGIC – IF YES, GO TO Q28i

i. Please explain why you would or wouldn’t recommend the Little Orange Book:

29. Do you think the revised edition of The Little Orange Book could be improved?

- Yes
- No
- Prefer not to say

LOGIC – IF YES, GO TO Q29i

i. If you selected yes, please explain how this could be improved:

30. What would be the most useful format for the revised edition of The Little Orange Book? Please click one.

- Hard copy
- Online
- Mobile phone app
- Other (please state)
- Prefer not to say

i. Please explain your answer:

# PAGE 6: VIEW OF THE REVISED EDITION OF THE LITTLE ORANGE BOOK IF NOT RECEIVED BEFORE

We have put some pictures of the Revised Edition of The Little Orange Book below. The images below focus on advice about conjunctivitis which is one example of the advice provided by The Little Orange Book.


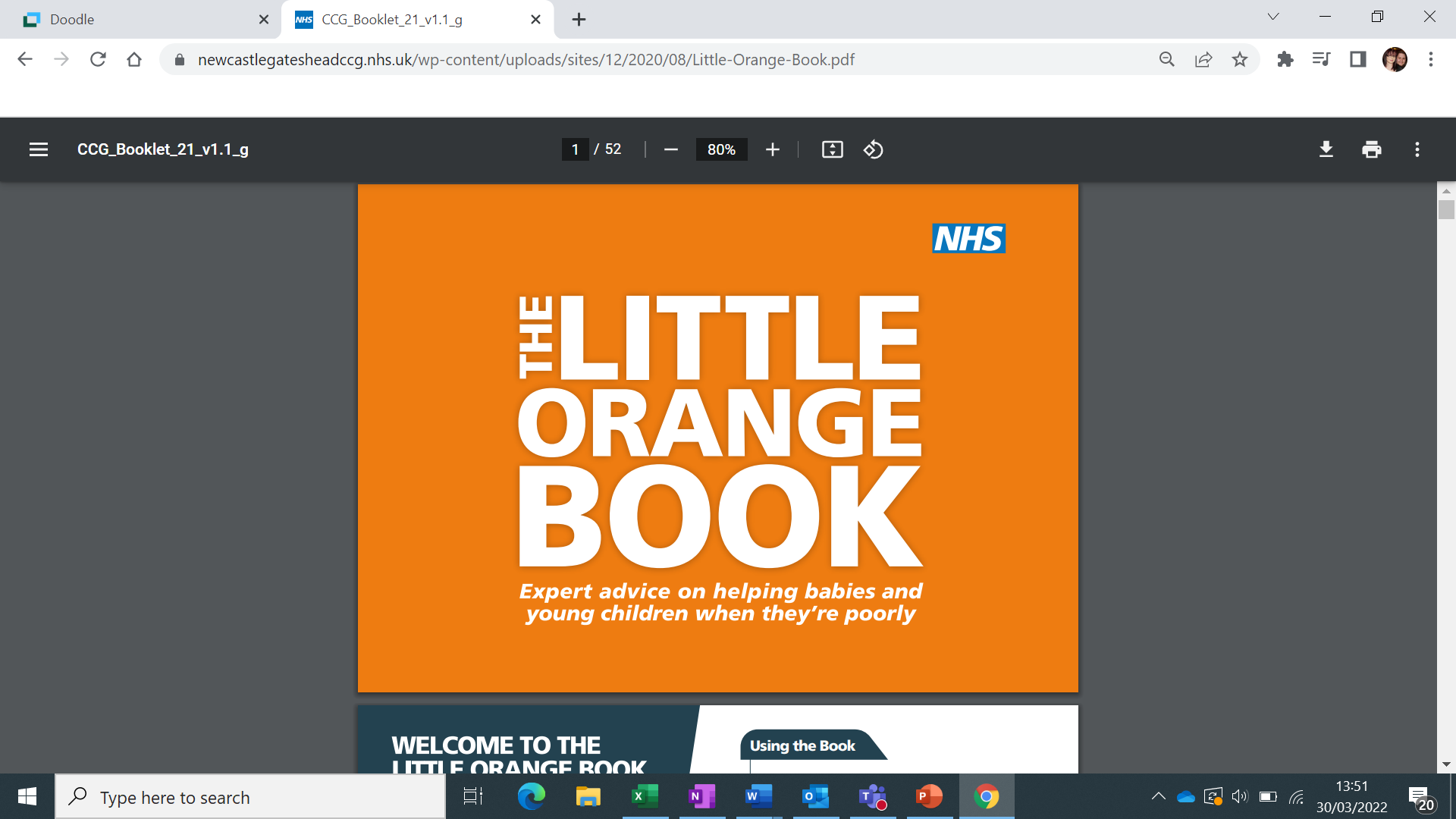


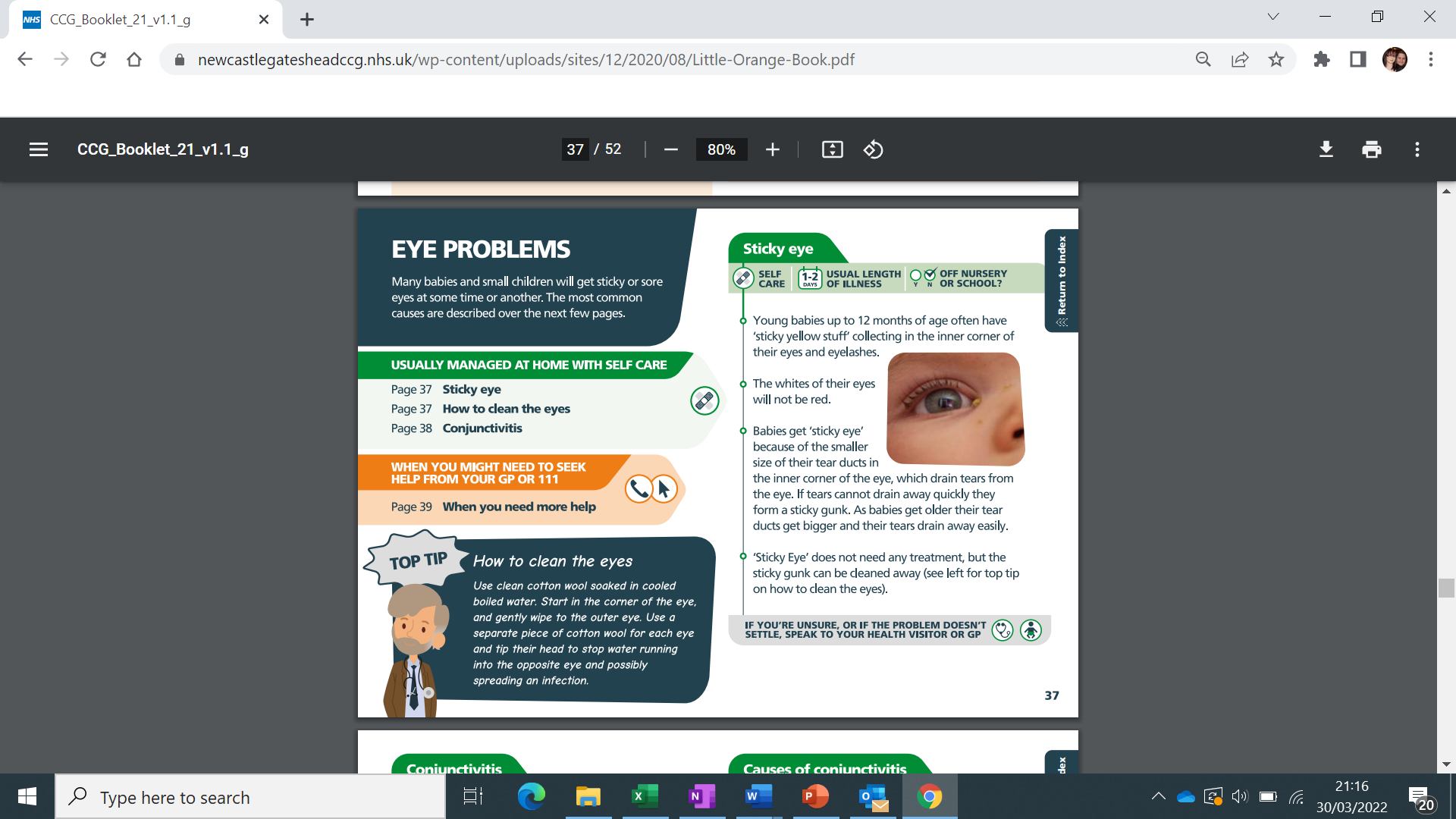


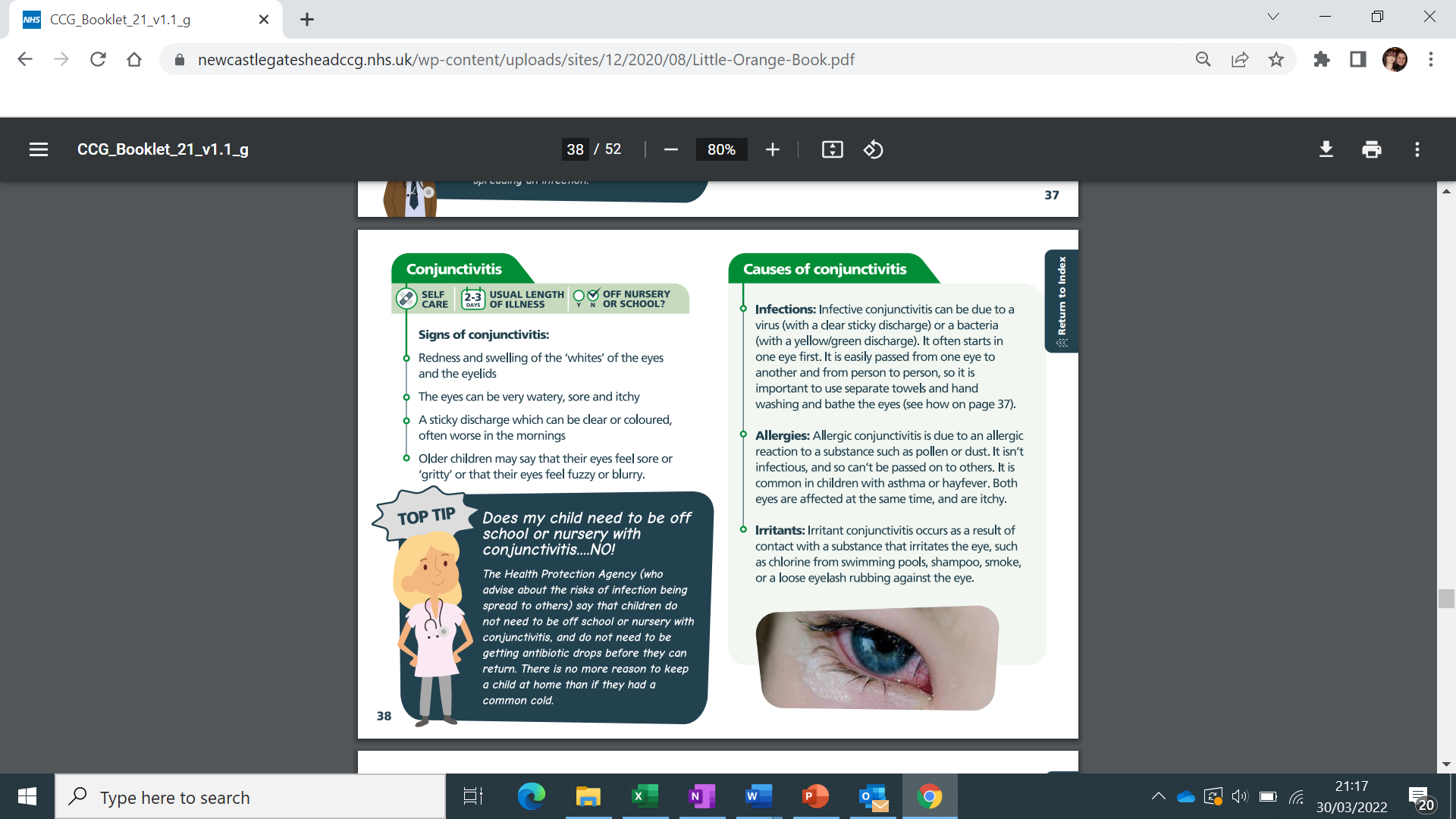


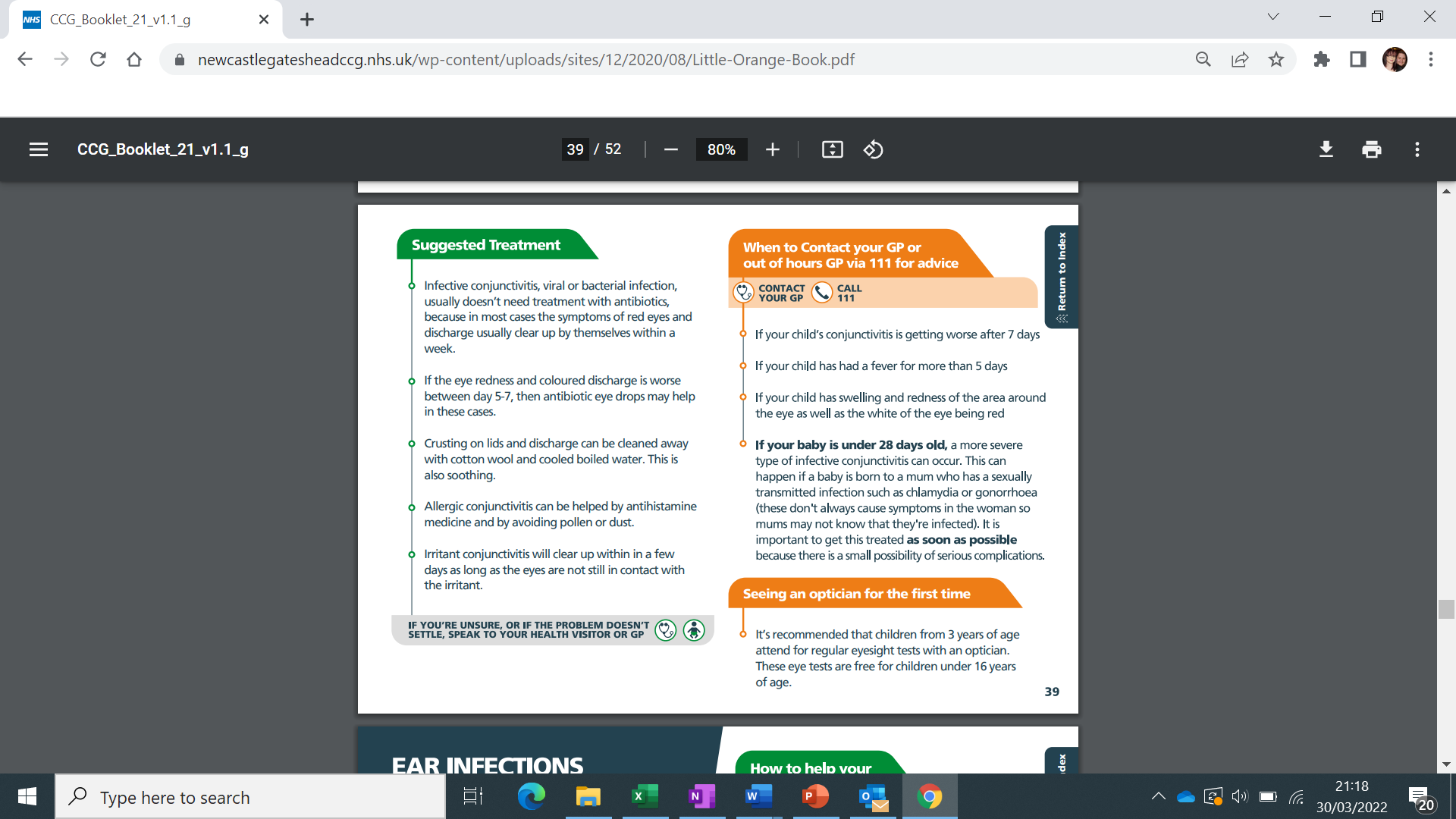


31. Do you think you would use this booklet to help you make decisions about caring for your child/children when they’re ill? (drop down menu)

- Yes
- No
- Prefer not to say

LOGIC – IF YES, MOVE ONTO Q31i. IF NO, MOVE ONTO Q31ii

i. If yes, when do you think you would use this book to help you make decisions about caring for your child/children when they’re ill? (Open response)

ii. can you tell us why not? (Open response)

32. What information would you like to see in this book to help you make decisions for your child/children when they’re ill? (Open response)

33. How would you like this information presented? (e.g. layout, pictures) (Open response)

34. What would be the best way to get this book to you? (Drop down menu)

- GP surgery
- Walk in centre
- Urgent care
- Midwife
- Health visitor
- Children Centre
- Hospital (e.g. A & E)
- Online
- Through a mobile phone app
- Other (please state)
- Prefer not to say

# PAGE 7: USE OF HEALTHCARE SERVICES

35. When making a decision about your child’s/children’s health, where do you go for information? Please click all that apply (drop down menu)

- Family members (e.g. parents)
- Online information
- Midwife
- Health Visitor
- GP surgery
- Walk in centre
- A&E
- The Little Orange Book (Before 2018)
- The revised version of The Little Orange Book (after 2018)

36. Have you used health care services for your child/children in the past year? (drop down menu)

- Yes
- No
- Prefer not to say

*LOGIC – IF YES, GO TO 36i. IF NO, GO TO Q37

i. If yes, what services have you used? (drop down menu)

- Midwife
- Health visitor
- GP surgery
- Walk in centre
- Urgent Care
- A&E
- Other (please state)
- Prefer not to say

*LOGIC – BASED ON RESPONSES TO THE ABOVE QUESTION, USER IS DIRECTED TO THE NEXT QUESTION. FOR INSTANCE, IF SELECTED GP AND MIDWIFE, THEY WILL BE ASKED ABOUT THESE. FOR PREFER NOT TO SAY, GO TO Q37.

a. How many times have you contacted or seen a Midwife for your child/children? (drop down menu)

- None
- 1-2
- 3-4
- 5-7
- 8 or more

b. How many times have you contacted or seen a Health Visitor for your child/children? (drop down menu)

- None
- 1-2
- 3-4
- 5-7
- 8 or more

c. How many times have you contacted or seen the GP surgery for your child/children? (drop down menu)

- None
- 1-2
- 3-4
- 5-7
- 8 or more

d. How many times have you been to a walk in centre for your child/children? (drop down menu)

- None
- 1-2
- 3-4
- 5-7
- 8 or more

e. How many times have you contacted or went to urgent care for your child/children? (drop down menu)

- None
- 1-2
- 3-4
- 5-7
- 8 or more

f. How many times have you went to A&E for your child/children? (drop down menu)

- None
- 1-2
- 3-4
- 5-7
- 8 or more

ii. Why did you access these services? (open response)

iii. Thinking of your child/children whom you accessed services for, do they have any long-term health issue that need regular health care appointments? (drop down menu)

- Yes
- No
- Prefer not to say

*LOGIC – IF YES, GO TO 36iiia. IF NO, GO TO 36iv

a. If yes, and you feel comfortable doing so, can you please say what this condition(s) is below? Please include for each child with a long-term health condition(s). (open response)

# PAGE 8: COVID-19 PANDEMIC

37. Did you use the revised edition of the Little Orange Book during the COVID-19 pandemic and Nationwide lockdowns?

- Yes
- No
- Prefer not to say

*LOGIC – IF NO GO TO PAGE 9. IF YES, GO TO Q38

38. Which of the following services did you use during the COVID-19 pandemic and Nationwide lockdowns?

- GP surgery
- Walk in centre
- Urgent care
- A&E
- Midwife
- Health visitor
- Other (please state)
- Prefer not to say

Please rate the extent that you agree with the following statements:

39. The COVID-19 restrictions affected my decision to access healthcare for my child/children

- Strongly agree
- Agree
- Neither agree nor disagree
- Disagree
- Strongly disagree

40. The COVID-19 restrictions changed my usual way of accessing healthcare services

- Strongly agree
- Agree
- Neither agree nor disagree
- Disagree
- Strongly disagree

41. I used the revised edition of The Little Orange Book more during the COVID-19 restrictions

- Strongly agree
- Agree
- Neither agree nor disagree
- Disagree
- Strongly disagree

42. I was more reluctant to visit health services during the COVID-19 restrictions

- Strongly agree
- Agree
- Neither agree nor disagree
- Disagree
- Strongly disagree

43. When my child is unwell, I tend to use the internet to get advice and information

- Strongly agree
- Agree
- Neither agree nor disagree
- Disagree
- Strongly disagree

# PAGE 9: INFORMATION ABOUT YOU

We would like to ask you some questions about you. This information helps us to understand who is using the Revised Version of The Little Orange Book and how we can improve it. The data we collect will be anonymised and will not be identifiable.

45. What is your gender? (drop down menu)

- Male
- Female
- Non-binary/Third Gender
- Other (please state)
- Prefer not to say

47. What is your ethnic group? Please select only ONE category from the list below (drop down menu)

- White
  - White: White English/Welsh/Scottish/Northern Irish/British
  - White: Irish
  - White: Gypsy/Traveller or Irish Traveller
  - White: Any other white background
- Mixed/Multiple Ethnic Groups
  - Mixed/Multiple Ethnic Groups: White and Black Caribbean
  - Mixed/Multiple Ethnic Groups: White and Black African
  - Mixed/Multiple Ethnic Groups: White and Asian
  - Mixed/Multiple Ethnic Groups: Any other mixed/multiple ethnic background
- Asian/Asian British
  - Asian/Asian British: Indian
  - Asian/Asian/British: Pakistani
  - Asian/Asian British: Bangladeshi
  - Asian/Asian British: Chinese
  - Asian/Asian British: Any other Asian background
- Black/African/Caribbean/Black British
  - Black/African/Caribbean/Black British: African
  - Black/African/Caribbean/Black British: Caribbean
  - Black/African/Caribbean/Black British: Any other Black, African, Caribbean background
- Other ethnic group
  - Other ethnic group: Arab
  - Other ethnic group: Any other ethnic group
- Prefer not to say.

48. How old are you? (open response)

49. What is your annual household income (per year)? (drop down menu income brackets)

- Less than £20,000
- £20,000 - £30,000
- £30,000 - £40,000
- £40,000 - £50,000
- £50,000 - £60,000
- £60,000 - £70,000
- More than £70,000
- Prefer not to say

50. What is the highest level of educational qualification you have? (drop down menu)

- - No qualifications
  - Level 1 (e.g. BTEC/NVQ)
  - Level 2 (e.g. GCSE/O level/BTEC/NVQ)
  - Level 3 (e.g. AS level/BTEC/NVQ)
  - Level 4/5 Undergraduate Degree/Diploma (e.g. University Foundation Level)
  - Level 6 Bachelor's Degree (e.g. University BSc, BA)
  - Level 7 Master's Degree or postgraduate degree (e.g. MA, MSc)
  - Doctorate (e.g. PhD)
  - Other
  - Prefer not to say

51. What is your employment status? (drop down menu)

- Employed full time (at least 35 hours per week)
- Employed part time (under 34 hours per week)
- Self employed
- Unpaid/Voluntary work
- Homemaker/Housewife/Househusband
- Student
- Carer
- Retired
- Unemployed currently looking for work
- Unemployed not currently looking for work
- Unable to work
- Other (please state)
- Prefer not to say

52. What is your relationship status? (drop down menu)

- Single
- Married/Civil partnership/Co-inhabiting
- Separated
- Divorced
- Widowed
- Other (please state)
- Prefer not to say

53. Do you have any long-term physical or mental health condition(s) or illnesses? (drop down menu)

- Yes
- No
- Prefer not to say

*LOGIC – IF YES, GO TO 53i. IF NO, GO TO Q54

i. If yes, and you feel comfortable doing so, can you please state below what your long-term physical or mental health condition(s) or illness is: open response)

54. Is English your first language? (drop down menu)

- Yes
- No
- Prefer not to say

*LOGIC – IF YES, GO TO 54i. IF NO, GO TO Q55

i. If not, what is your first language? (open response)

55. I feel confident reading information or books (drop down menu)

- Strongly agree
- Agree
- Neither agree nor disagree
- Disagree
- Strongly disagree
- Prefer not to say

# PAGE 10: INFORMATION ABOUT YOUR CHILD/CHILDREN

We would like to ask you some questions about your child/children. We collect this information to learn more about the people who are taking part in this survey and to help us see which groups we may need to ask in the future. The data we collect will be anonymised and will not be identifiable.

56. Are you a first-time parent/carer?

- Yes
- No
- Prefer not to say

LOGIC – IF NO, GO TO Q56i, IF YES GO TO Q57

i. Do you have more than one child?

- Yes
- No
- Prefer not to say

LOGIC – IF YES, GO TO 56i. IF NO, GO TO Q57

ii. How many children do you have? (drop down menu)

- One
- Two
- Three
- Four
- More than four
- Prefer not to say

iii. Is there a gap of more than 5 years between your youngest child and your older children? (drop down menu)

- Yes
- No
- Prefer not to say

iv. Do you feel that having more than one child with an age gap between them has affected your knowledge and confidence regarding when to access healthcare services? Please explain. (open response)

57. Have you had a child since 2018? (drop down menu)

- Yes
- No
- Prefer not say

58. What are the living arrangements of your child/children? (drop down menu)

- Live with you full-time
- Live with you part-time (e.g. Coparenting/shared custody)
- Live with other family members
- In foster care
- Other (please state)
- Prefer not to say

# PAGE 11: PARTICIPATING IN FURTHER RESEARCH

59. How did you hear about this survey?

- Through my GP surgery (e.g. text message, flier)
- Social media
- Word of mouth
- Other (please state)
- Prefer not to say

60. Would you like to take part in an interview about your views and experiences using the revised edition of The Little Orange Book?

- Yes
- No

*LOGIC – IF YES, GO TO 61. IF NOT, GO TO PAGE 12.

61. Would you prefer to take part in an individual interview or a focus group about the revised edition of The Little Orange Book?

- Individual interview (one-to-one interview with a member of the research team)
- Focus group (interview with other parents/carers and a member of the research team)

*LOGIC – IF FOCUS GROUP IS SELECTED, MOVE TO Q62. IF INDIVIDUAL INTERVIEW IS SELECTED MOVE TO Q63.

62. The focus group will take place over Microsoft Teams. Please choose one of the following dates.

- 10:30am 19^th^ April 2022 (Gateshead residents)
- 10:30am 29^th^ April 2022 (Newcastle residents)
- 1pm 22^nd^ April 2022 (Gateshead residents)
- 1pm 2^nd^ May 2022 (Newcastle residents)

63. Please enter your name, preferred method of contact (e.g. email address or telephone number) and preferred time to be contacted:

A member of the research team will be in touch to organise an interview with you or provide you with the information for the focus group.

# PAGE 12: VOUCHER COMPETITION ENTRY

Thank you for taking part in the survey about the Revised Edition of The Little Orange Book. We are offering everyone who takes part the chance to be entered into a prize draw to win a Highstreet voucher (worth £25). If you would like to be entered into this draw, please provide your email address or contact telephone number below.

The information that you provide here will not be included with the answers you have given for this survey.

We will notify the winner by 1^st^ June 2022.

# PAGE 13: THANK YOU AND DEBRIEF STATEMENT

Thank you for your participation in the survey. Your responses have been received.

If you have any concerns following completion of the survey, please address any queries you may have to: Dr Lynette Shotton ([lynette.shotton@northumbria.ac.uk](mailto:lynette.shotton@northumbria.ac.uk)) or Dr Amy Johnson ([amy6.johnson@northumbria.ac.uk](mailto:amy6.johnson@northumbria.ac.uk)).

The questions and content within this survey may have raised emotional issues. If you feel you need support and have been affected by any issues raised within the survey, please find details of a charity that you can contact here:

Samaritans / Telephone: 116 123 (UK) / Website: www.samaritans.org You are able to call Samaritans for free, at any time, from any phone.

If you have immediate concerns about your wellbeing, please seek medical attention from your general practitioner or alternative health care provider. If you require out of hours medical care, you are able to contact NHS 111 for assistance or dial 999 in an emergency situation.

If you have any concerns about the study and would like to talk to someone at the University of Northumbria who knows about, but is not involved in, the study please contact: Dr Se Kwang Hwang ([sekwang.hwang@northumbria.ac.uk](mailto:sekwang.hwang@northumbria.ac.uk)).

You can access the online version of The Little Orange Book through this link: <https://newcastlegatesheadccg.nhs.uk/wp-content/uploads/sites/12/2020/08/Little-Orange-Book.pdf>
