# Supplementary material for: “The impact of the Little Orange Book on how parents/carers manage symptoms of illness in children: A mixed methods study”
Source: PLoS One. 2025 Jan 22;20(1):e0295470. doi: 10.1371/journal.pone.0295470 (PMC11753658; doi:10.1371/journal.pone.0295470)
Supplement: Attachment — Submitted filename: Research Ethics 41385.pdf [file pone.0295470.s002.pdf]

My Documents

Submission

Submission Ref 41385

Status New

Submission Coordinator

Name: Lynette Shotton — Lynette Shotton

Email: lynette.shotton@northumbria.ac.uk

Faculty: Health and Life Sciences

Department: Social Work, Educ & Comm Wellbeing

Submitting As: Staff

Externally Approved: ☐ Note: ONLY tick this box if your project has already received full ethical approval from an external organisation

Module Level Approval: ☐ *Tick this box if staff and this submission refers to an entire module.*

** Only to be used for low or medium risk projects as categorised by the diagnostic risk question set **

Module Code: *Type a value* Help

Module Tutor: Find Help Clear

Titl...

De...

Em...

Research Supervisor: Find Help Clear

Titl...

De...

Em...

Ethical Risk Level: High

Click here to answer the ethical risk questions

Ethical Risk Diagnostic Questions and Responses

Refresh

ID	QUESTION	ANSWER
	No items to display.	

Co-investigators

Add Edit Delete Save Refresh

NAME OF CO-INVESTIGATORS

Matthew Breckons (Newcastle University)

Kathryn Carruthers (Teesside University)

Professor Judith Rankin (Newcastle University)

Floor Christie De Jong (Sunderland University)

Sunil Bhopal (Newcastle University)

G1: General Aims and Research Design (Mandatory)

Title

Title of your research project

Experiences and perspectives of parents and carers on the revised Little Orange Book: An evaluation

Outline General Aims and Research Objectives

State your research aims/questions (maximum 500 words). This should provide the theoretical context within which the work is placed, and should include an evidence-based background, justification for the research, clearly stated hypotheses (if appropriate) and creative enquiry.

Brief background
The Little Orange Book (LOB) was developed by Newcastle Gateshead Clinical Commissioning Group (NGCCG) in 2016 and aimed to help parents and carers of young children to self-manage common childhood symptoms and to encourage appropriate use of health services. While a previous evaluation has suggested that the LOB was meeting its aims, following updating of the book (in 2018) and the Covid-19 pandemic in which patterns of health service use have changed dramatically, it is important to understand the role of the LOB in this new healthcare landscape.

AIM AND OBJECTIVES
Aim
Building on and updating previous evaluation work, the aim of this work is to understand parent/carer views of the revised LOB since its introduction in 2018.
Objectives
1. To identify a sample of parents / carers from each geographic area (Gateshead and Newcastle) who have used the revised LOB since 2018,
2. To identify if, and how parents / carers use the book, what they liked about it, barriers to use and if/how it could be improved,
3. To explore any perceived impacts of the book, for example behaviour change of parents, increased confidence to manage health conditions and use of health services,
4. To consider 2) and 3) in the context of the Covid-19 pandemic any changes to how health services are accessed,
5. To identify suggestions for further development of the book,
6. To make recommendations for future research.

G2: Research Activities (Mandatory)

Please give a detailed description of your research activities

Please provide a description of the study design, methodology (e.g. quantitative, qualitative, practice based), the sampling strategy, methods of data collection (e.g. survey, interview, experiment, observation, participatory), and analysis. Do sensitive topics such as trauma, bereavement, drug use, child abuse, pornography, extremism or radicalisation inform the research? If so have these been fully addressed?

Data collection will comprise: 1) an online survey (with offline and brief completion options), and 2) qualitative focus groups and interviews.
Public Involvement and Community Engagement (PICE)
We will establish a small group of parents (n=2-3) from existing local parent networks or public involvement organisations who we will engage with formally during study set up, at the dissemination phase and on an ad-hoc basis throughout the study. We will aim to engage with communities often under-represented in research with reference to the NIHR INCLUDE guidance. We will engage with the NENC ARC Public Involvement and Community Engagement (PICE) lead in order to obtain advice and access to existing networks.

1) LOB service user feedback survey
An online survey will be developed using freely available Jisc online survey software. The survey will capture both quantitative and qualitative (free text) responses.

Recruitment
Distribution of this survey will be via key contacts at a number of organisations that have distributed the LOB; these include both health services in the CCGs mentioned and a range of education and community services in the areas of Gateshead and Newcastle for parents. The survey will be publicised by fliers and posters and targeted social media posts containing a URL and QR link to the survey, as well as contact information of the research team for those wishing to take part who do not have digital access and would like to request a hard-copy of the survey. At these venues, we will also leave copies of a hard copy feedback card as a brief option which will ask three key questions:
(1) How have you used the LOB?; (2) How did it help you?; (3) How can we improve it?.

We will invite those who complete the survey to participate in either a group or an individual telephone interview with the research team via the research team's contact details which will be provided.

Data collection
The survey will capture a range of demographic information from survey participants including but not limited to age, gender, ethnicity, annual household income, religion, whether the parent and/or child are people living with a disability, the age and number of children, household members, geographical location, and level of educational attainment, whether any children in the household have a long-term condition.

Multiple choice survey questions will explore awareness and use of the LOB, perceived impact on decision-making regarding the child's symptoms and subsequent health care use, accessibility and preferred formats for information. Use of conditional questions will allow relevant follow-up free-text questions which will explore respondents' views on the content of the LOB, facilitators and barriers to use and suggested amendments.

M1: People and/or Personal Data

☑ **Tick if your work involves people and/or personal data?**

Sample Groups

Provide details of the sample groups that will be involved in the study and include details of their location (whether recruited in the UK or from abroad) and any organisational affiliation. For most research studies, this will cover: the number of sample groups; the size of each sample group; the criteria that will be used to select the sample group(s) (e.g. gender, age, sexuality, health conditions). If the sample will include NHS staff or patients please state this clearly. If this is a pilot study and the composition of the sample has not yet been confirmed, please provide as many details as possible.

The participants will be parents / carers who were previously or are currently living in the Newcastle and Gateshead area who have used the Little Orange Book from 2018 to date. Several thousand copies of the Little Orange Book have been disseminated and the survey will aim to capture as many of the parents / carers as possible. From the respondents we will seek to interview either in group or individual interviews 10 parents / carers from each location (20 in total). We will sample purposively based on demographics such as age, sex, number of children, ethnic background, educational attainment, use of healthcare services and perceptions of the LOB and our approach will be informed by NIHR INCLUDE guidance, reference to regional demographics and under-served groups; seeking advice from NENC ARC PICE representatives where necessary. Our sampling strategy may be updated based on preliminary analysis of data.
The participants must be able to provide consent to take part in the study.
Participants must be a minimum of 16 years of age.

Nature of data pertaining to Living Individuals

If you will be including personal data of living individuals, including still or moving images, please specify the nature of this data, and (if appropriate) include details of the relevant individuals who have provided permission to utilise this data, upload evidence of these permissions in the supporting documentation section.

Details of any Special Category Data - If you will be collecting data revealing racial or ethnic origin, political opinions, religious or philosophical beliefs, trade union membership, data concerning health or data concerning a natural person's sex life or sexual orientation, please specify which categories you will be using.

Survey data will be captured from a sample of parents in the Newcastle and Gateshead CCG localities who have used the LOB. Their survey data will be anonymised. Data collected will seek to obtain personal characteristics which will include racial or ethnic origin. This is to ascertain uptake across the CCG population and to inform future developments of the LOB to improve it and make it more accessible to all parents within these communities. A sub-section of those completing the survey will be invited to a combination of group and individual interviews. All data collected through these methods will be anonymised. During group interviews - if face to face is possible- the participants may know each other, but guidelines will be in place asking for the participants to respect each others confidentiality. Any data collected will be anonymised by the research team. Group interviews held virtually will afford participants the choice of revealing their identity on camera / using a pseudonym. The team will use Teams to comply with UNN regulations relating to remote working and data collection.

Legal Basis for Processing: Further guidance can be found here

If you require further information, please contact the Data Protection Officer by emailing

dp.officer@northumbria.ac.uk

Participants in this study will be given information about what data is captured - they will be able to choose what data they share with the research tea. Their data will be anonymised and stored safely on university password protected devices / computers. Any transcipts will be anonymised and any reports and publications will not name participants. The data will be stored for the duration of the study (approximately 6 months) and for up to a further 18 months to facilitate scholarly publications.
Northumbria University GDPR regulations will be followed. Equally, the research team comprises experienced researchers from across the North East who will also comply with GDPR regulations. As above - individual and group interviews, where it is not possible to hold face to face, will be conducted via Teams with permission to record from participants. Only voice recordings will be obtained and this will be made clear to the participants.

Recruitment

Describe the step by step process of how you will contact and recruit your research sample and name any organisations or groups that will be approached. Your recruitment strategy must be appropriate to the research study and the sensitivity of the subject area. You must have received written permission from any organisations or groups before you begin recruiting participants. Copies of draft requests for organisational consent must be included in the 'Supporting Documentary Evidence'. You must also provide copies of any recruitment emails/posters that will be used in your study.

The CCGs have data recording where they have distributed copies of the LOB. This includes a range of health locations across Newcastle and Gateshead CCG, as well as education settings in these areas and community groups. These services will be used as a way to promote the study: The first stage of the evaluation is a survey will be publicised by fliers and posters and targeted social media posts containing a URL and QR link to the survey, as well as contact information of the research team for those wishing to take part who do not have digital access and would like to request a hard-copy of the survey. At these venues, we will also leave copies of a hard copy feedback card as a brief option which will ask three key questions: (1) How have you used the LOB?; (2) How did it help you?; (3) How can we improve it?.

☐ Remuneration

Details of remuneration

Will you make any payment or remuneration to participants or their carers/consultees? If yes: Please provide details/justifications. Note that your Faculty may have specific guidelines on participant payments/payment rates etc and you should consult these where appropriate.

Participants taking part in individual and group interviews will be given a small token/ voucher to cover the costs of their travel and time. This will amount to no more than £15 per participant

Type of Consent

Informed Consent

Type of Consent Details

Please include copies of information sheets and consent forms in the 'G6: File Attachments' section. If the study involves participants who lack capacity to consent, procedures in line with sections 30-33 of the Mental Capacity Act will need to be put in place. If you are using alternative formats to provide information and /or record consent (e.g. images, video or audio recording), provide brief details and outline the justification for this approach and the uses to which it will be put:

A study information / consent sheet is attached in this submission.

Researcher and Participant Safety Issues

If there any risks the research could cause any discomfort or distress to participants (physical, psychological or emotional) describe the measures that will be put in place to alleviate or minimise them. Please give detailsof the support that will be available for any participants who become distressed during their involvement with the research.

Those taking part in the study will do so freely. The PIs contact details will be available to participants and the PI will be available to talk through any issues. The evaluation focuses on those within Newcastle and Gateshead CCGs and health services in these areas will be a further source of support if needed.

Data Gathering Materials Used

Provide a detailed description of what the participants will be asked to do for the research study, including details about the process of data collection (e.g. completing how many interviews / assessments, when, for how long, with whom). Add any relevant documentation to the 'Supporting Documentary Evidence' section of this form.

Participants will be asked to complete one survey, which can be completed either electronically or via a hard copy. This will take no more than 30 minutes. Hard copy feedback cards will be left at venues within the two CCGs and these will take about 5 minutes to complete. Those who have completed these will be asked if they would like to take part in either 1 individual or 1 group interview. Those taking part will be asked to give up to 1 hour of their time to complete the interview. The interviews will be conducted by the research team. Alongside the team already identified we plan to recruit an experienced research assistant who will lead on data collection with support of the research team.

Potential Ethical Issues

Please describe any potential ethical issues the project may have which are not covered above, and how you have sought to minimise these.

The project seeks to voluntarily capture the views of parents who have used the LOB as part of this service evaluation. It is not anticipated that participation will cause distress, however, should this occur the research team will be available for debriefing. The PI is an experienced nurse and health visitor and as such, has expertise in supporting parents. The research team will also support parents and signpost to relevant sources of support within the CCGs i.e. health visitors, GP. should this be needed.

H1: Children (i.e. under 18s) and Vulnerable Adults

☐ Tick if your work involves children or vulnerable adults?

M2: DBS Clearances Required

Add Edit Delete Save Refresh

Do not upload your DBS certificate to this system as this would be contravening General Data Protection Regulations.

Further information relating to DBS Clearance can be found in the Ethics and Governance Handbook using the link below

Ethics and Governance Handbook

********* All fields below relating to DBS certificates must be completed *********

NAME OF PERSON ON CERTIFICATE	TYPE OF DBS CLEARANCE	CERTIFICATE REFERENCE	ADULTS/CHILDREN	DATE OF DBS CERTIFICATE

(Add new row)

M3: Secondary Data

☐ Tick if you will be using secondary data NOT in the public domain?

M4: Commercial Data

☐ Tick if your work involves commercially sensitive data?

M5: Environmental Data

☐ Tick if your work involves the collection of environmental data?

H2: Human Tissue

☐ Tick if your work involves use of human tissue?

H3: Animal Subjects

☐

Tick if your work involves the use of animal subjects (excluding abattoir derived material and commercially sourced fur)?

H4: Security Sensitive Information (including Prevent-related material)

☐ Do you require access to material that is prohibited/restricted (e.g. under Government security classifications or the Official Secrets Act)?

H5: Cultural Sensitivity

☐ Tick if your work involves Art or Artefacts (including culturally sensitive items?)

H6: Reason for resuming face to face research with human participants during Covid-19

Please outline the justification for commencing the research, balancing the risk with the potential benefits of the research, and with reference to the principles that underpin considerations around re-starting research?

• Research should only restart/ start when safe to do so. The safety of research participants and personnel is of paramount importance. If it is possible to undertake the research remotely, this is the preferred option.

• The research must be able to be conducted in a way that aligns with local government guidance as a minimum. The robustness of these requirements in promoting safeguarding should also be considered and exceeded if needed.

• The research will not require that the researcher or participants puts themselves at any greater risk than they would otherwise pertain as they conduct their daily lives within guidelines set out by the government at the time.

Type a value

H7: Research Viability and Capacity during Covid-19

Is this research project feasible from a scientific, financial, or practical point of view during the Covid-19 pandemic?

• For sponsored studies within NHS/ Social care there is an expectation that Sponsors, and sites will work together to conduct a preliminary assessment of all paused studies.

• Delivery of research might be dependent on collaborator, their local arrangements and policies and capacity to undertake the study within the necessary timescales. e.g., health and care services affected by COVID-19 in different ways. It is important for researchers to understand and agree readiness of collaborators and sites where this is essential to the delivery of the research activity.

• Delivery of research must be possible considering the study's population size, proposed location and its cap...

The evaluation is funded by the CCGs and will include funding to recruit an RA who will be involved in developing the survey, data collection, analysis and production of two reports (one for each CCG). Alongside this some time has been costed in for the PI and the other members of the team are providing in kind support. The team have already had several meetings to develop and successfully win the bid for this evaluation and are confident it is feasible. The team have experience in conducting similar evaluation projects and undertaking these during the pandemic. The evaluation has already been fully costed by UNN.

G3: Research Data Management Plan (Mandatory)

Anonymising Data (mandatory)

Describe the arrangements for anonymising data and if not appropriate explain why this is and how it is covered in the informed consent obtained.

Survey data will not ask for names / key personal identifiers such as date of birth. Surveys will have a unique code. Those participants wishing to take part in either a group or individual interview will be asked to contact the PI. For individual and group interviews each participant will be given a pseudonym / unique number.

Storage Details (mandatory)

Describe the arrangements for the secure transport and storage of data collected and used during the study. You should explain what kind of storage you intend to use, e.g. cloud-based, portable hard drive, USB stick, and the protocols in place to keep the data secure.

If you have identified the requirement to collect 'Special category data', please specify any additional security arrangements you will use to keep this data secure.

Survey data will be stored electronically using JISC, which is accepted by UNN. Any data relating to survey responses will be stored via university password protected online systems. Should hard copies of the survey be completed these will be anonymised, scanned and stored electronically and the hard copy destroyed immediately after this via confidential waste processes.
It is not anticipated that interviews will be face to face, but should this occur, portable equipment (voice recorders) data will be downloaded and erased as soon as possible following the recording and stored safely by the research team. No personal identifying data will be captured.
It is expected that Teams will be used to conduct the interviews and voice recording from these will be stored via university cloud based storage systems.

Retention and Disposal (mandatory)

☑ **I confirm that I will comply with the University's data retention schedule and guidance.**

Research Data Management link

General Data Protection Regulations including Data Protection link

Records Retention Schedule link

G4: Research Project Timescale (Mandatory)

Proposed Start Date	04/01/2022
Proposed End Date	30/06/2022

G5: Additional Information

☑ Externally Funded

External Funder: Other - please provide details of funder below

Please give details of your 'other' funder

Newcastle Gatesgead Clinical Commissioning Group

Agresso Reference

Type a value

☐ Franchise Programme Organisation

Please give details of your franchise organisation

Type a value

☑ NHS Involvement

Please give details of any NHS involvement

Type a value

☐ Clinical Trial(s)

Please give details of any Clinical Trial(s)

Type a value

☐ Medicinal Products

Please give details of any Medicinal Product(s)

G6: File Attachments

Additional files can be uploaded e.g. consent documentation, participant information sheet, etc.

Please note: It is best practice to combine all documents into one PDF (This avoids the reviewer having to op...

Go To Attachments

G7: Health and Safety (Mandatory)

- [x] I confirm that I have read and understood the University's Health and Safety Policy.
- [x] I confirm that I have read and understood the University's requirements for the mandatory completion of risk assessments in advance of any activity involving potential physical risk.

The University Health and Safety Policy can be accessed here

The University Risk Assessment Code of Practice can be accessed here

Please confirm either:

- [ ] There are PHYSICAL risks associated with the research project work and I confirm that a risk assessment has been approved and attached to this ethics submission.

OR

- [x] I can confirm that there are no physical risks associated with this project and so no risk assessments are required.

Students requiring assistance with completing their risk assessment should get in touch with their supervisor or module tutor as the first point of contact. If further assistance is needed, the Faculty Technician can provide further guidance.

For more specific risk assessments (e.g. lab work), especially where the project is Medium or High risk, you are required to consult the Faculty Technical Manager; your Supervisor/Module Tutor will be able to put you in touch.

If you have any questions or concerns, please contact the University Health and Safety Team by emailing

CRHealthandSafety@northumbria.ac.uk

G8: Insurance (Mandatory)

- [x] I have read and understood the University Insurance guidance document (link below):

Insurance Guidance link

If you think your activity may involve a High Risk rating or are unsure how to answer the statements - contact fi.insurance@northumbria.ac.uk with a copy of your research proposal for advice.

I confirm my work is covered by University Insurance. I confirm an insurance risk level of:

Low

If your insurance risk level is HIGH please attach details of exceptional insurance coverage:

Click here to attach a file

G9: Electronic Signature (Mandatory)

- [ ] I confirm my supervisor has reviewed the contents of this document
- [ ] I confirm I have assessed the ethical risk level of my work correctly and answered the above sections as fully and accurately as possible.

Full Name

Date

PDF Version

Create PDF

No items to display.

Review Comments, Conditions and Outcomes

Log of any Ethical Incidents

Log New Incident

INCIDEN...	CREATED DATE TIME	CREATOR NAME	COMPLAINANT DETAILS

No items to display.

Title and Objectives (see G1)

Add Save

Reviewer A: Reviewer B:

e.g. Are the research question and/or study aims clear?

DATE	ROLE	COMMENT

No items to display.

Proposed Methodology and Analysis (see G2)

Add Save

Reviewer A: Reviewer B:

e.g. Is the design appropriate to the research question?
Are the methods of data analysis appropriate to the research question?

DATE	ROLE	COMMENT
No items to display.		

Sample and Recruitment (see M1)

Add Save

Reviewer A: Reviewer B:

e.g. Is the sampling approach appropriate to the design?
Is the sample sufficient and achievable?
Is the process of recruitment clearly explained?
Are participants receiving payments for taking part, and if so is the payment appropriate?
If the DBS is ticked, has the appropriate information been included?

DATE	ROLE	COMMENT
No items to display.		

Consent (see M1)

Add Save

Reviewer A: Reviewer B:

e.g. Is the approach to consent seeking clear?
Is consent from parents/ carers/ guardians required?
Are all necessary recruitment and informed consent documentation included (e.g. letters of permission, letters of invitation)
Is the information sheet adequate to ensure informed consent?
Are the consent form(s) appropriate?

DATE	ROLE	COMMENT
No items to display.		

Researcher and Participant Safety (see M1)

Add Save

Reviewer A: Reviewer B:

e.g. Is there any risk of physical harm for the researcher(s) or the participants and if so what attempts have been made to alleviate or minimise them?
Have Risk Assessments been referred to where appropriate?

DATE	ROLE	COMMENT
No items to display.		

Research Activities (see G2-G8, M1-M5, H1-H5)

Add Save

e.g. Are the research tasks described clearly?
Do sensitive topics such as trauma, bereavement, drug use, child abuse, pornography or extremism/ radicalism inform the

research? If so have these been fully addressed? (and we can use this to amend the information on risk levels on the form)Is there any risk that the tasks may cause psychological harm and if so what attempts have been made to alleviate or minimise them?

DATE	ROLE	COMMENT
	No items to display.	

Data Management Plan (see G3)

Add Save

Reviewer A: **Reviewer B:**

e.g. Have sufficient steps been taken to ensure participant anonymity/confidentiality of data?
Are the arrangements for data storage and disposal clearly outlined?
Are these arrangements in line with University and/or the funding body requirements?

DATE	ROLE	COMMENT
	No items to display.	

File Attachments (see G6)

Add Save

Please note: where file attachments have not been added because they are not required, please select Approve.

COMMENT BY	DATE	ROLE	COMMENT
	No items to display.		

General Comments (see Help)

Add Save

DATE	ROLE	COMMENT
	No items to display.	
