# Supplementary material for: “The impact of the Little Orange Book on how parents/carers manage symptoms of illness in children: A mixed methods study”
Source: PLoS One. 2025 Jan 22;20(1):e0295470. doi: 10.1371/journal.pone.0295470 (PMC11753658; doi:10.1371/journal.pone.0295470)
Supplement: Attachment — Submitted filename: Reviewer responses.docx [file pone.0295470.s003.docx]

| Coach Lane Campus  Northumbria University  15 Coach Lane  Newcastle upon Tyne  NE7 7XA    Dear Reviewers at Plos One    Please find our revised manuscript entitled “The impact of the Little Orange Book on how parents/carers manage symptoms of illness in children: A mixed methods study”. Thank you for reviewing our submission and for providing detailed feedback. We have considered each of the points in turn and the table below provides detail of how we have responded. We do hope this is now acceptable.  Please address all correspondence to Dr Lynette Shotton ([lynette.shotton@northumbria.ac.uk](mailto:lynette.shotton@northumbria.ac.uk)). Thank you for considering this manuscript and we look forward to your response.    Yours Sincerely  Lynette Shotton, Amy Johnson, Kathryn Carruthers and Matthew Breckons | | | | |
| --- | --- | --- | --- | --- |
| 1. | Ensure meets PlosOne style requirements | [https://journals.plos.org/plosone/s/file?id=wjVg/PLOSOne_formatting_sample_main_body.pdf](https://eur02.safelinks.protection.outlook.com/?url=https%3A%2F%2Fjournals.plos.org%2Fplosone%2Fs%2Ffile%3Fid%3DwjVg%2FPLOSOne_formatting_sample_main_body.pdf&data=05%7C02%7Clynette.shotton%40northumbria.ac.uk%7Cb54278d576f64e22790f08dc48a7760e%7Ce757cfdd1f354457af8f7c9c6b1437e3%7C0%7C0%7C638465131437466183%7CUnknown%7CTWFpbGZsb3d8eyJWIjoiMC4wLjAwMDAiLCJQIjoiV2luMzIiLCJBTiI6Ik1haWwiLCJXVCI6Mn0%3D%7C0%7C%7C%7C&sdata=u9IJJi8xSImcWHeQkXDkZFL6n5TbXAdfyEsMTo5beV8%3D&reserved=0) and  [https://journals.plos.org/plosone/s/file?id=ba62/PLOSOne_formatting_sample_title_authors_affiliations.pdf](https://eur02.safelinks.protection.outlook.com/?url=https%3A%2F%2Fjournals.plos.org%2Fplosone%2Fs%2Ffile%3Fid%3Dba62%2FPLOSOne_formatting_sample_title_authors_affiliations.pdf&data=05%7C02%7Clynette.shotton%40northumbria.ac.uk%7Cb54278d576f64e22790f08dc48a7760e%7Ce757cfdd1f354457af8f7c9c6b1437e3%7C0%7C0%7C638465131437470754%7CUnknown%7CTWFpbGZsb3d8eyJWIjoiMC4wLjAwMDAiLCJQIjoiV2luMzIiLCJBTiI6Ik1haWwiLCJXVCI6Mn0%3D%7C0%7C%7C%7C&sdata=dYJh3HjEc4JK7vc3FepgMkJAZ9ZcMNMiM8BYs0%2FXinA%3D&reserved=0). |  |  |
| 2. | Please provide additional details re participant consent | In the ethics statement in the Methods and online submission information, please ensure that you have specified (1) whether consent was informed and (2) what type you obtained (for instance, written or verbal, and if verbal, how it was documented and witnessed). If your study included minors, state whether you obtained consent from parents or guardians. If the need for consent was waived by the ethics committee, please include this information. |  | LS this has been added to page 6 (online survey section) and page 7 interview phase. |
| 3. | Please state what role the funders had | If the funders had no role, please state: ""The funders had no role in study design, data collection and analysis, decision to publish, or preparation of the manuscript.""  If this statement is not correct you must amend it as needed.  Please include this amended Role of Funder statement in your cover letter; we will change the online submission form on your behalf. |  | Added to the Financial Disclosure Statement |
| 4. |  | Please provide a complete Data Availability Statement in the submission form, ensuring you include all necessary access information or a reason for why you are unable to make your data freely accessible. If your research concerns only data provided within your submission, please write "All data are in the manuscript and/or supporting information files" as your Data Availability Statement. |  | Uploaded |
| 5. | Please include captions for your Supporting Information files at the end of your manuscript, and update any in-text citations to match accordingly. | Please see our Supporting Information guidelines for more information: [http://journals.plos.org/plosone/s/supporting-information](https://eur02.safelinks.protection.outlook.com/?url=http%3A%2F%2Fjournals.plos.org%2Fplosone%2Fs%2Fsupporting-information&data=05%7C02%7Clynette.shotton%40northumbria.ac.uk%7Cb54278d576f64e22790f08dc48a7760e%7Ce757cfdd1f354457af8f7c9c6b1437e3%7C0%7C0%7C638465131437481890%7CUnknown%7CTWFpbGZsb3d8eyJWIjoiMC4wLjAwMDAiLCJQIjoiV2luMzIiLCJBTiI6Ik1haWwiLCJXVCI6Mn0%3D%7C0%7C%7C%7C&sdata=yrpc2iuLxPfcTY7mKGBgnMA5J0qFzANMDpa3NjINVDA%3D&reserved=0). |  | This has been amended |
| Comments to authors |  |  |  |  |
| 1 | Proof read |  |  | Proof reading has taken place. |
| 2. | Abstract | Make sure objectives and aim is the same as the intro  Change perceptions to experiences  Results – review numbers of participants and include in method and not results  The abstract would benefit from being re-written. It doesn’t align well with the paper, especially the objectives. | In line with reviewer's comments, the abstract has been rewritten. |  |
| 3 | Intro | Line 50 out of context – improve flow of into and funnel towards aim.  Is there a secondary aim  Reduce length of aim i.e aim to understand parent/carer views and experiences of using LOB  You write a lot about the previous evaluation of LOB, perhaps you would have benefited from clarifying everything about it in the introduction.  Some of the existing literature around this topic is referred to, albeit it is brief and doesn’t provide a clear overview. I would suggest amending the first sentence for clarity, perhaps: ‘For parents and carers of young children, making decisions about when and where to access health care or services when they are acutely unwell is complex.’ I don’t understand what you mean by ‘research highlights difficulties in making appropriate decisions’ – are you referring to parents having difficulty making these decisions? If so, say that. ‘A recent systematic review suggested that pre-disposing factors (socioeconomic status, ethnicity and race) may influence parental decisions on the use of care which are often based on perceptions of the urgency of the condition [3]’ – These ‘pre-disposing factors’ are just a small part of the picture and this systematic review made far wider and valuable contributions to the evidence base around parental decision-making in accessing unscheduled care. I suggest re-visiting this source and providing a more comprehensive and accurate overview of the main messages. ‘Prompted by dramatic reductions in the use of children’s health services during the Covid-19 pandemic….’ If you are going to make a strong statement like this, you will need to cite the quantitative data source which tells you there were ‘dramatic reductions’ in paediatric access to health services. ‘While health information has the potential to cause anxiety [5].’ – Incomplete sentence. ‘The resource aims to support parents/carers (< 5 years of age)’ – Please re-phrase as it sounds like the parents are less than five years of age. Please be clearer about the aim of this paper and, importantly, it differs to what was published in the evaluation. This is fundamental to satisfy the key criteria of this journal i) whether the study presented the results of original research and ii) whether the results reported have not been published elsewhere. | Thank you for these comments. We have made the suggested changes to our wording to provide clarification. We have added further references to support the reduction in the use of children’s healthcare services. We have also sought to provide clarity about a previous evaluation within the introduction, as well as highlighting the value of our current evaluation.  We agree that the systematic review provided a valuable contribution to understanding in this complex area – we have added a (brief) summary of the other findings from this review.  We have amended the punctuation with regards to reference [5] and the age of parents.  We have amended the text regarding the aims of the study to clarify the aims of this manuscript. |  |
| 4. | Design | Line 74 – clarify the two phases consist of a quant and qual part  Line 78-82 – move to intro or improve relevance  Line 82-83 – give ethics its own heading and add more about how consent was obtained and how confidentiality was maintained | Methods section amended in response to comments. |  |
|  | Participants | Criteria for inclusion / exclusion are lacking – make sure the info is consistent i.e. if we exclude those who have not received LOB why include them in the survey? What sampling methods were used? How was ssamples size calculated  Table are hard to read – would prefer all text in one column and numbers in their own  Renove the column for those who did not revied the book as they are not included in the results  Line 103 states the numbers included in the analysis – use this consistently i.e. in the abstract | We have added more information about inclusion/exclusion criteria. We included the views of individuals who had not used the LOB as… These individuals were included in the overall analysis and are presented elsewhere. We could incorporate these views into the present paper, however this could appear ‘messy’.  The tables have been amended in response to reviewer comments. |  |
|  | Online survey | I would have liked more information about your survey. For example; which one have you used, how is it created, is it tested and validated? One suggestion is that you attach the survey or put the questions in a box. If the survey was made specifically for this study, please clarify this. Line 115: that your participants could win money for their participation should preferably be placed in the ethics section. Interviews  How did the online survey inform the qualitative interview component – this needs better explained. Please use an appropriate reporting tool for qualitative interview studies (ie COREQ) Where is the interview topic guide? This needs to be included as a supplementary file. ‘All respondents noted their use of health services for their child in the previous year, as well as during the Covid-19 pandemic.’ – this requires an explanation. | The online survey allowed purposive sampling in the qualitative phase as well as allowing us to explore trends in data (through focused questions); we have added further detail regarding this. |  |
|  | Interviews | You have chosen to do both individual and group interviews, why? Here you also change the method when you include three people via word of mouth, why? In general, I am having trouble understanding how your interviews/group interviews were conducted, please attach your interview guide. This is also something you should consider including in your method-discussion and clarify how it may have affected the results. |  | This section has been reviewed and detail about this phase has been improved. |
|  | Data analysis | This section needs to be developed, as it is now it is not possible for me as a reader to understand how and what you have done. The analysis process needs to be described. The descriptive analysus part also needs to be developed, eg what measures were used and were there any internal loss?  Much greater detail on the process of thematic analysis is required. | We have added further description to the qualitative analysis section, we hope that this provides clarification of our process. |  |
|  | Results | It is difficult to assess the trustworthiness of the results because the data-analysis is not properly described. In general; the result is not perceived as fully analyzed. There are repetitions and similar results are under several headings. The quotes get more space than the result itself. I would have liked a table to illustrate the quantitative results, especially since it’s not specified in the method section which questions were asked.  Please explain how you have integrated the survey and interview findings – without a clearer explanation of your methods earlier, it is difficult to follow the narrative of your findings and, more importantly, to have confidence in the trustworthiness of the data. Further in-depth analysis is needed for all themes. The findings are very descriptive with little text and an overuse of exemplar quotations. | We have added to the methods which we hope provides further clarity and supports our results better. |  |
|  | Discussion / reccommendations | Could be divided under two headings for easier reading. Your discussion is interesting and well-formulated, perhaps I would have liked the discussion to be based on more references. Missing a clear method discussion that includes concepts such as trustworthiness; dependability, confirmability, credibility, transferability (qualitative research) and validity and reliability (quantitative research).  The discussion is interesting and with further integration of the literature allowing comparison and contrasts with your findings and the existing evidence base, it would be much stronger. You need to further consider some of the limitation in your work. |  |  |
